# Myoglobin adsorption and saturation kinetics of the cytokine adsorber Cytosorb® in patients with severe rhabdomyolysis: a prospective trial

**DOI:** 10.1186/s13613-024-01334-x

**Published:** 2024-06-22

**Authors:** Helen Graf, Caroline Gräfe, Mathias Bruegel, Michael Zoller, Nils Maciuga, Sandra Frank, Lorenz Weidhase, Michael Paal, Christina Scharf

**Affiliations:** 1 Department of Anaesthesiology, LMU University Hospital, LMU Munich, Munich, Germany; 2 Institute of Laboratory Medicine, LMU University Hospital, LMU Munich, Munich, Germany; 3 Medical Intensive Care Unit, University Hospital Leipzig, Leipzig, Saxony, Germany

**Keywords:** Rhabdomyolysis, Myoglobin, Cytosorb, Myoglobin clearance, Adsorption, Renal replacement therapy

## Abstract

**Background:**

Rhabdomyolysis is a serious condition that can lead to acute kidney injury with the need of renal replacement therapy (RRT). The cytokine adsorber Cytosorb® (CS) can be used for extracorporeal myoglobin elimination in patients with rhabdomyolysis. However, data on adsorption capacity and saturation kinetics are still missing.

**Methods:**

The prospective Cyto-SOLVE study (NCT04913298) included 20 intensive care unit patients with severe rhabdomyolysis (plasma myoglobin > 5000 ng/ml), RRT due to acute kidney injury and the use of CS for myoglobin elimination. Myoglobin and creatine kinase (CK) were measured in the patient´s blood and pre- and post-CS at defined time points (ten minutes, one, three, six, and twelve hours after initiation). We calculated Relative Change (RC, %) with: $1 - \left( {concentration(pre - post)\,/\,concentration\left( {pre} \right)} \right)*100$. Myoglobin plasma clearances (ml/min) were calculated with: $\left( {bloodflow*\left( {1 - hematocrit} \right)} \right)*\left( {concentration\left( {pre - post} \right)\,/\,concentration\left( {pre} \right)} \right)$

**Results:**

There was a significant decrease of the myoglobin plasma concentration six hours after installation of CS (median (IQR) 56,894 ng/ml (11,544; 102,737 ng/ml) vs. 40,125 ng/ml (7879; 75,638 ng/ml) (*p < 0.001*). No significant change was observed after twelve hours. Significant extracorporeal adsorption of myoglobin can be seen at all time points (*p < 0.05*) (ten minutes, one, three, six, and twelve hours after initiation). The median (IQR) RC of myoglobin at the above-mentioned time points was − 79.2% (-85.1; -47.1%), -34.7% (-42.7;-18.4%), -16.1% (-22.1; -9.4%), -8.3% (-7.5; -1.3%), and − 3.9% (-3.9; -1.3%), respectively. The median myoglobin plasma clearance ten minutes after starting CS treatment was 64.0 ml/min (58.6; 73.5 ml/min), decreasing rapidly to 29.1 ml/min (26.5; 36.1 ml/min), 16.1 ml/min (11.9; 22.5 ml/min), 7.9 ml/min (5.5; 12.5 ml/min), and 3.7 ml/min (2.4; 6.4 ml/min) after one, three, six, and twelve hours, respectively.

**Conclusion:**

The Cytosorb® adsorber effectively eliminates myoglobin. However, the adsorption capacity decreased rapidly after about three hours, resulting in reduced effectiveness. Early change of the adsorber in patients with severe rhabdomyolysis might increase the efficacy. The clinical benefit should be investigated in further clinical trials.

**Trial registration:**

ClinicalTrials.gov NCT04913298. Registered 07 May 2021, https//clinicaltrials.gov/study/NCT04913298.

**Graphical Abstract:**

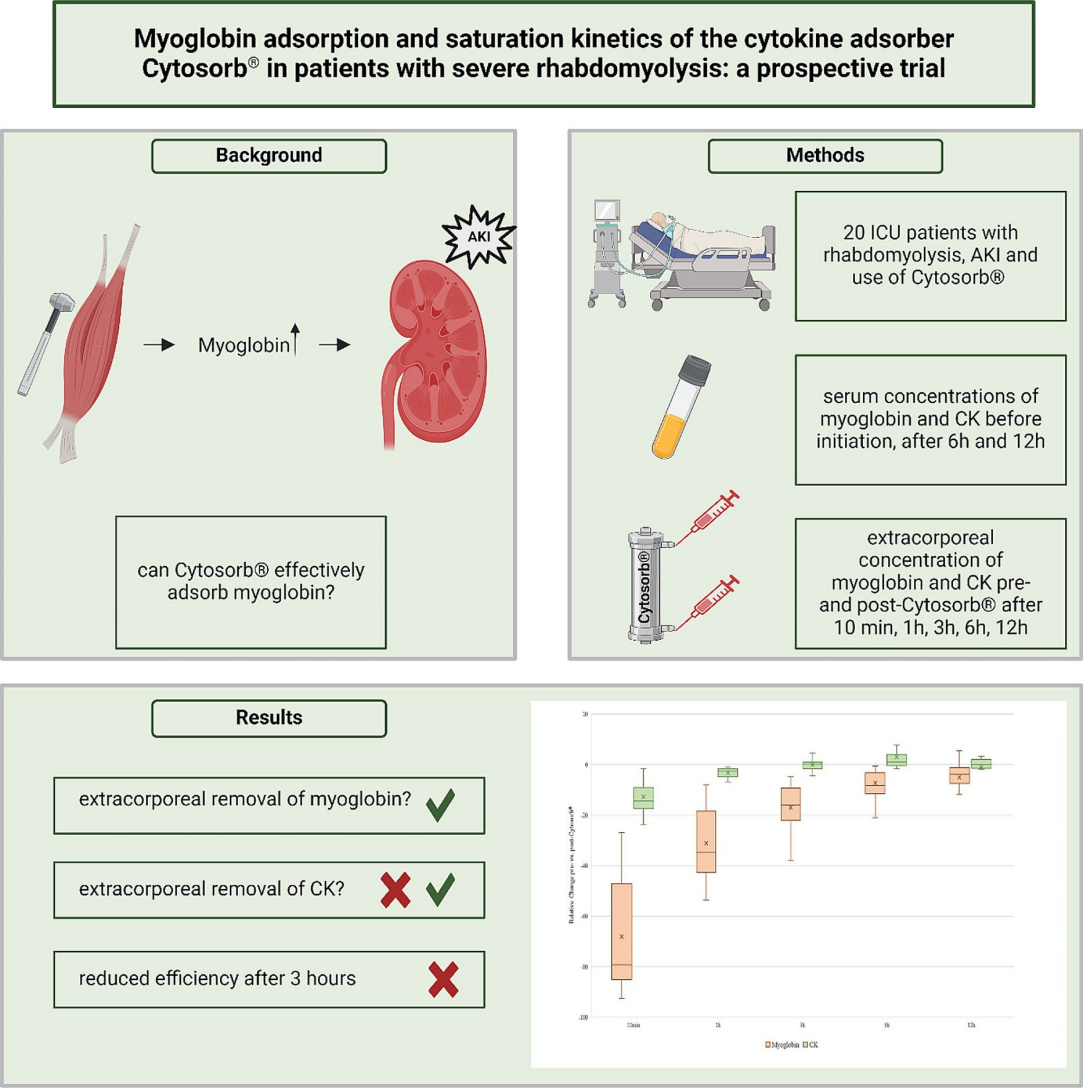

**Supplementary Information:**

The online version contains supplementary material available at 10.1186/s13613-024-01334-x.

## Introduction

Rhabdomyolysis is a serious condition characterized by muscle damage and lysis of skeletal muscle cells, which can lead to acute and chronic kidney injury, electrolyte disorder, hypovolemia and acidosis [1–3].

A triad out of elevation of creatine kinase (CK), myalgia /muscle swelling and brown (tea-colored) urine can be observed in affected patients. Since there is no established definition for rhabdomyolysis, it is characteristically diagnosed by an increase of CK five times above normal [4]. The triggers for rhabdomyolysis are diverse. It is most frequently caused by muscle damage by trauma, vascular obliteration or physical overexertion. Furthermore, infections and sepsis, drugs, hypokalemia, or hereditary muscle disorders can lead to rhabdomyolysis [1, 5]. Due to muscle damage, high levels of CK, myoglobin, urea, potassium, and other organic acids are released into the bloodstream, causing acidosis, arrhythmias, and hemodynamic instability [6]. Another severe complication of rhabdomyolysis is acute kidney injury (AKI), which occurrs in up to 50% of patients and may require renal replacement therapy [2, 5, 7]. Beyond, the occurrence of AKI in patients with rhabdomyolysis is associated with a poorer prognosis and higher mortality [1, 8–10].

The pathomechanism of AKI caused by rhabdomyolysis is complex and not quite recognized. Myoglobin can precipitate with the Tamm-Horsfall protein, particularly if the urine is acidotic and occludes the renal tubule systems as a result [11, 12]. Further, direct renal toxicity of myoglobin due to the oxidation of hydroxyl radicals has been described [2, 5]. Third, myoglobin has a vasoconstrictive effect on renal arterioles and intravascular volume depletion leads to aggravation of AKI [1, 2, 13].

To date, there is no specific therapy for rhabdomyolysis. In addition to treating the cause of rhabdomyolysis, therapy includes adequate volume therapy, application of diuretics, urine alkalization and, if necessary, RRT. Since myoglobin seems to be the main cause of kidney damage in rhabdomyolysis, one therapeutic approach is extracorporeal removal from the bloodstream. A few studies and case reports already shown successful elimination of myoglobin through the use of a high-flux dialyzer or a medium-/ high cut-off dialyzer [14–16]. Another option is to use the cytokine adsorber Cytosorb**®** (CS). CS eliminates hydrophobic molecules up to a size of 55 kDa and is approved for the removal of cytokines, bilirubin, myoglobin, ticagrelor and rivaroxaban. It is largely used in patients in hyperinflammatory conditions like sepsis [17]. There are still few data describing the use of CS in patients with rhabdomyolysis. Dilken et al. presented a significant reduction of myoglobin (17 kDa) and CK, even though the CK with a size of 80 kDa should be beyond the adsorption spectrum [18]. Scharf et al. also showed a significant decrease of myoglobin by CS in a retrospective study of 43 patients with severe rhabdomyolysis [19].

To date, we do not have any information about the actual adsorption capacity and saturation kinetics of Cytosorb**®** for myoglobin. However, this information is of considerable relevance to ensure targeted therapy for these patients. Therefore, we conducted a prospective study to evaluate the adsorption performance and saturation kinetics of CS for myoglobin and CK. In addition, differences in the CS clearance regarding to initial concentration of myoglobin are another point of interest. The aim is to be able to assess the appropriate duration of use and to be able to suggest changing intervals for effective therapy.

## Methods

### Study setting

This is a single-center, prospective observational exploratory study to investigate the adsorption rate and saturation kinetics of CS for myoglobin and CK in patients with severe rhabdomyolysis. The patients were included between May 2021 and August 2022 during their stay in two intensive care units (ICU) at the Ludwig-Maximilians-University hospital in Munich. The local institutional review board approved the study (registration number 21–0236). The study was registered with clinicaltrials (NCT04913298). Prior to inclusion in the study, written informed consent was obtained from patients or their legal representatives as approved by the review board.

### Study population

Adult patients (≥ 18 years) with the need for continuous RRT due to anuric/oliguric acute kidney injury, diagnosed according to the KDIGO consensus criteria, and CS-application, were included [20]. In addition, patients had to be diagnosed with rhabdomyolysis and plasma myoglobin levels > 5000 ng/ml. As mentioned above, the exclusion criterion was no consent from the patient or their legal representatives to participate in the study. The indication of CS application was at the discretion of the attending physician and independent of the study. As there was no previous data at the time of the study design, the number of cases to be included was set at 20 patients as an exploratory study. This was expected to capture potential variability in adsorption capacity. No sample size estimation was performed in the absence of available data.

### Blood sampling and characteristics of RRT

CS was installed post-dialyzer. The patients received treatment with continuous RRT with multiFiltrate® device. Either continuous veno-venous hemodialysis (CVVHD) with citrate anticoagulation (CiCa) with Ultraflux® AV 1000 S filter or a postdilution continuous veno-venous hemodiafiltration (CVVHDF) with Ultraflux® AV 600 S filter and heparin anticoagulation was used. Both filters have a cut-off of approximately 30 kDa [21]. Blood samples (EDTA tubes) were taken at the extracorporeal circuit directly before the cartridge (= pre-CS) and directly after the cartridge (= post-CS) at defined time points. The sampling times were ten minutes after the start of CS treatment, and one, three, six, and twelve hours after initiation. Furthermore, myoglobin and CK plasma levels were measured shortly before the initiation of CS and after six and twelve hours. EDTA anticoagulated plasma was obtained by centrifugation of whole blood in the intensive care unit immediately after sampling. The separated plasma samples were immediately frozen and stored stably at -80 °C until measurement.

### Laboratory measurements

Clinical chemistry parameters were tested in plasma using the standard clinical chemistry modular analyzer Cobas®8000 (Roche Diagnostics, Mannheim, Germany) at the institute of laboratory medicine. We tested Myoglobin using a specific electrochemiluminescence immunoassay and quantified CK using a kinetic assay covering all isoforms.

### Data collection

For data evaluation, demographic data and clinical and laboratory variables were collected from the laboratory and patient information system. Different laboratory parameters were measured shortly before CS initiation in the clinical routine.

### Statistical analysis

The statistical analysis was performed using IBM SPSS statistics (Version 29.0. IBM Corp., Armonk, NY, USA). A paired *T-test* with associated samples was used to compare the concentrations pre- and post-CS after testing a normal distribution of the studied parameters (*Shapiro-Wilk*-test). For variables without a normal distribution, the Wilcoxon-Test was performed. To compare differences in myoglobin elimination in patients with or without a very high baseline myoglobin (> 50.000 ng/ml), the *U-test* was used. The relative change (RC) of the parameters by CS at different time points was calculated using:

$$\eqalign{ Relative\, & Change\left( \% \right)\, = \,1\, \cr & - \left( {{{concentration(pre - post)} \over {concentration\left( {pre} \right)}}} \right)\,*100 \cr}$$

In addition, the myoglobin plasma clearances of CS was calculated using:

$$\eqalign{& Plasmaclearance\left( {{{ml} \over {min}}} \right)\, = \, \cr & \left( {bloodflow\,*\,\left( {1\, - \,hematocrit} \right)} \right)\,*\,\left( {{{concentration\,\left( {pre\, - \,post} \right)} \over {concentration\,\left( {pre} \right)}}} \right) \cr}$$

## Results

### Demographic and clinical data

A total of 20 patients were included in the exploratory study. The main diagnoses at admission to the ICU in 40% of the patients were major surgical procedures such as liver or lung transplants or vascular surgery. 25% of the patients were admitted due to an acute respiratory distress syndrome (ARDS). The main causes for rhabdomyolysis were sepsis (30%) and compartment syndrome (20%). In 25% of the patients, a specific reason for rhabdomyolysis remained unknown. There was no myocardial infarction in any of the patients. The median age was 52 years and 75% of patients were male. The Simplified Acute Physiology Score II (SAPS II) on the day of CS treatment was 78 points and the 28-days mortality was 50%. All patients were either anuric or oliguric. The median urine output during CS-treatment was 0 ml. In five patients, CS therapy was discontinued prematurely between six and twelve hours due to filter clotting (*n* = 3), death (*n* = 1) and change of dialysis modality (*n* = 1). Detailed patient characteristics can be found in Table 1.

**Table 1:** Patient characteristics and laboratory measurements

	*n* (%) or median [IQR]
Patient characteristics	
Age (years)	52 [40; 63]
Gender: male/female	15 (75) / 5 (25)
Weight (kg)	79 [71; 106]
Height (m)	1.77 [1.69; 1.85]
BMI (kg/m²)	25 [22; 33]
28-day mortality	10 (50)
SAPS II	78 [69; 90]
**Renal replacement therapy**	
CVVHD	14 (70)
CVVHDF (post-dilution)	6 (30)
Blood flow (ml/min)	100 [100; 200]
Dialysate flow (ml/h)	2000 [2000; 2600]
Substitute flow rate (ml/h)	2000 [2000; 2375]
Ultrafiltration flow rate (ml/h)	100 [50; 100]
Dialysate dosis (ml/kg/h)	25.0 [18.0; 37.5]
Urine output during treatment	0 [0; 20]
**Laboratory parameters before initiation of Cytosorb®**	
Myoglobin (ng/ml)	56,894 [11,544; 102,737]
CK (U/l)	14,822 [2565; 27,090]
Creatinine (mg/dl)	1.8 [1.5; 2.7]
Urea (mg/dl)	76 [51; 100]
Lactate (mmol/l)	2.5 [1.8; 5.4]
Bilirubin (mg/dl)	3.2 [1.8; 9.8]
LDH (U/l)	1153 [468; 2202]
Interleukin-6 (pg/ml)	226 [51; 1593]
CRP (mg/dl)	10.6 [5.5; 17]

Note: BMI: body mass index, ICU: intensive care unit, SAPS II: Simplified Acute Physiology Score, CK: creatine kinase, CVVHD(F): continuous veno-venous hemodialysis / hemodiafiltration, LDH: lactate dehydrogenase, CRP: C-reactive protein

### Myoglobin and CK plasma concentration before and during CS-application

**Fig. 1:**
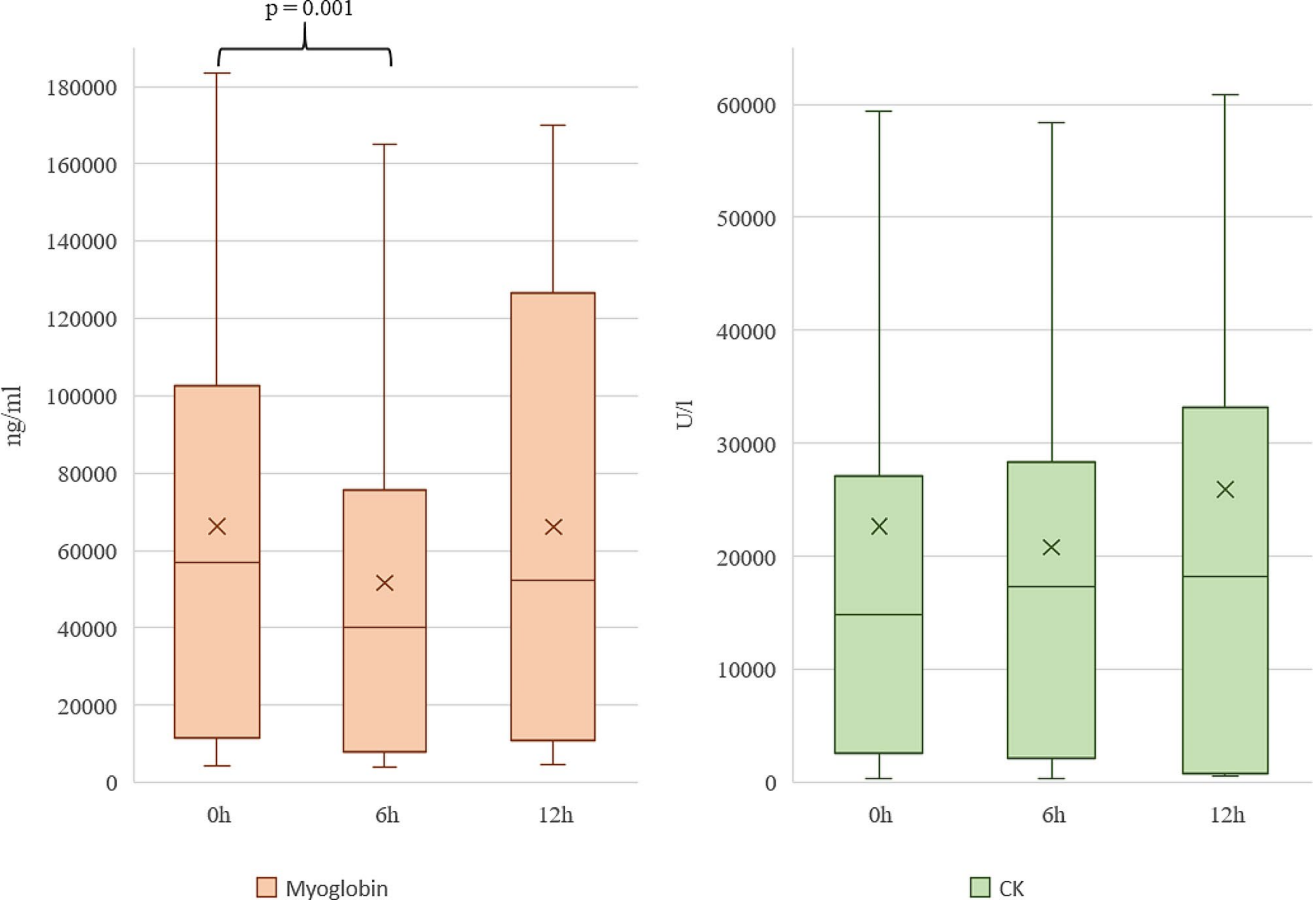
Plasma concentrations of myoglobin and CK at the defined time points. *Note * Plasma concentrations of myoglobin and CK before initiation, six and twelve hours after CS. The boxes of the boxplots represent the interquartile-range (IQR) and the line the median. Whiskers were limited to 1.5 times the IQR. The cross represents the mean

The median (IQR) myoglobin plasma concentration before initiation and at six and twelve hours was 56,894 ng/ml (11,544; 102,737 ng/ml), 40,125 ng/ml (7879; 75,638 ng/ml), and 52,189 ng/ml (10,848; 126,558 ng/ml), respectively. Six hours after the installation of CS, there was a significant decrease in plasma myoglobin levels (*p = 0.001*). No significant change was observed between six and twelve and between the initiation of CS and twelve hours. The median (IQR) CK plasma concentration at baseline and at six and twelve hours was 14,822 U/l (2565; 27,090 U/l), 17,333 U/l (2161; 28,328 U/l), and 18,275 U/l (831; 33,213 U/l), respectively. There was no significant change of CK plasma levels at any time. Figure 1 illustrates the myoglobin and CK plasma concentrations before, during, and at the end of CS application. Supplementary Table S1 presents the plasma concentrations of myoglobin (ng/ml) and CK (U/l) before initiation, six and twelve hours after CS of each patient.

### Extracorporeal elimination of myoglobin and CK

**Fig. 2:**
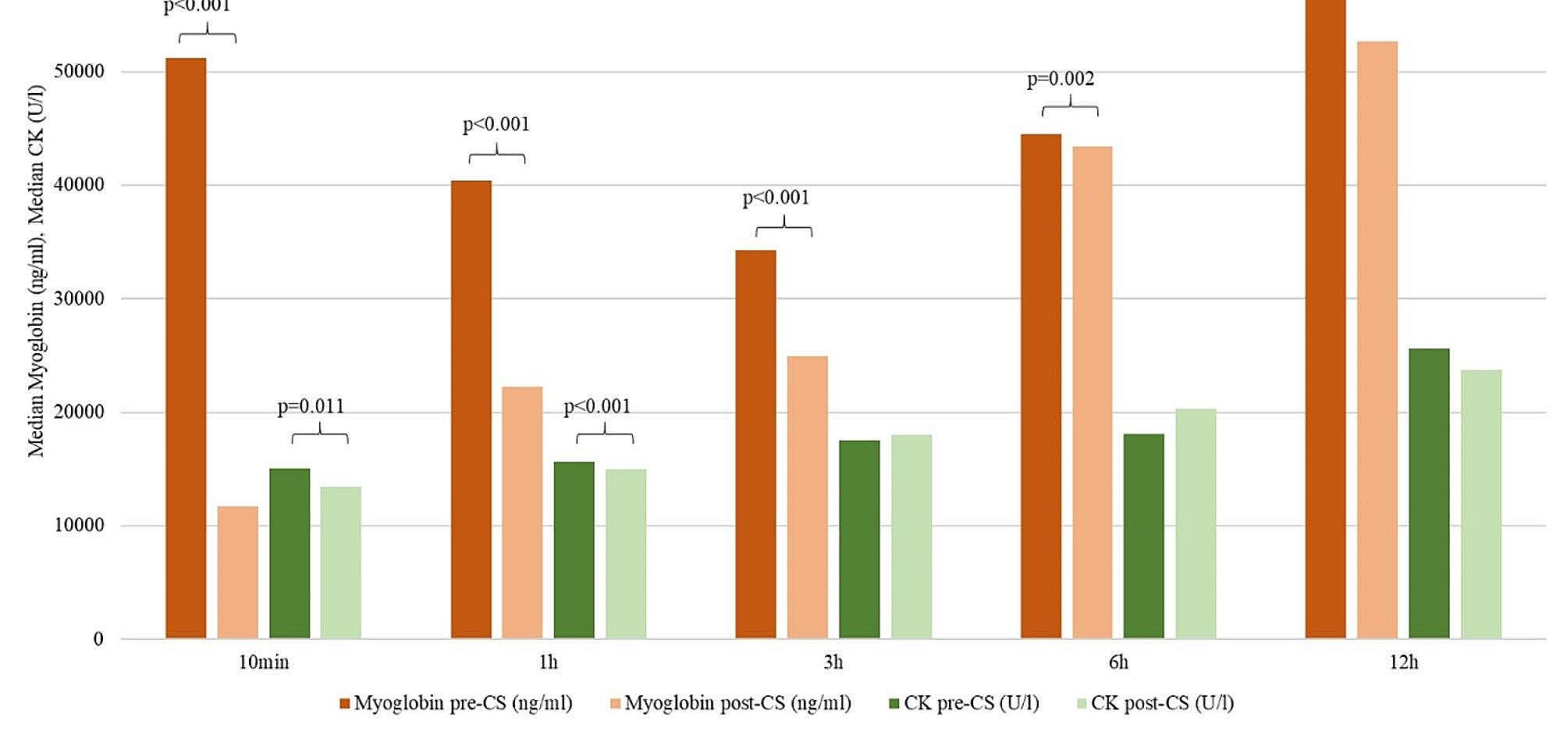
Median concentrations of myoglobin and CK pre- and post-CS

A significant (*p* < 0.05) extracorporeal reduction of myoglobin (pre-CS vs. post-CS) could be observed at all measured time points (ten minutes, one, three, six and twelve hours). There was also a significant decrease in CK at ten minutes (*p = 0.005*) and one hour after CS installation (*p < 0.001*). Figure 2 displays the median concentrations of myoglobin and CK pre- and post-CS at the defined time points. Supplementary Tables 2–3 present myoglobin (ng/ml) and CK (U/l) concentrations pre- and post-CS at the defined time points of each patient.

**Fig. 3:**
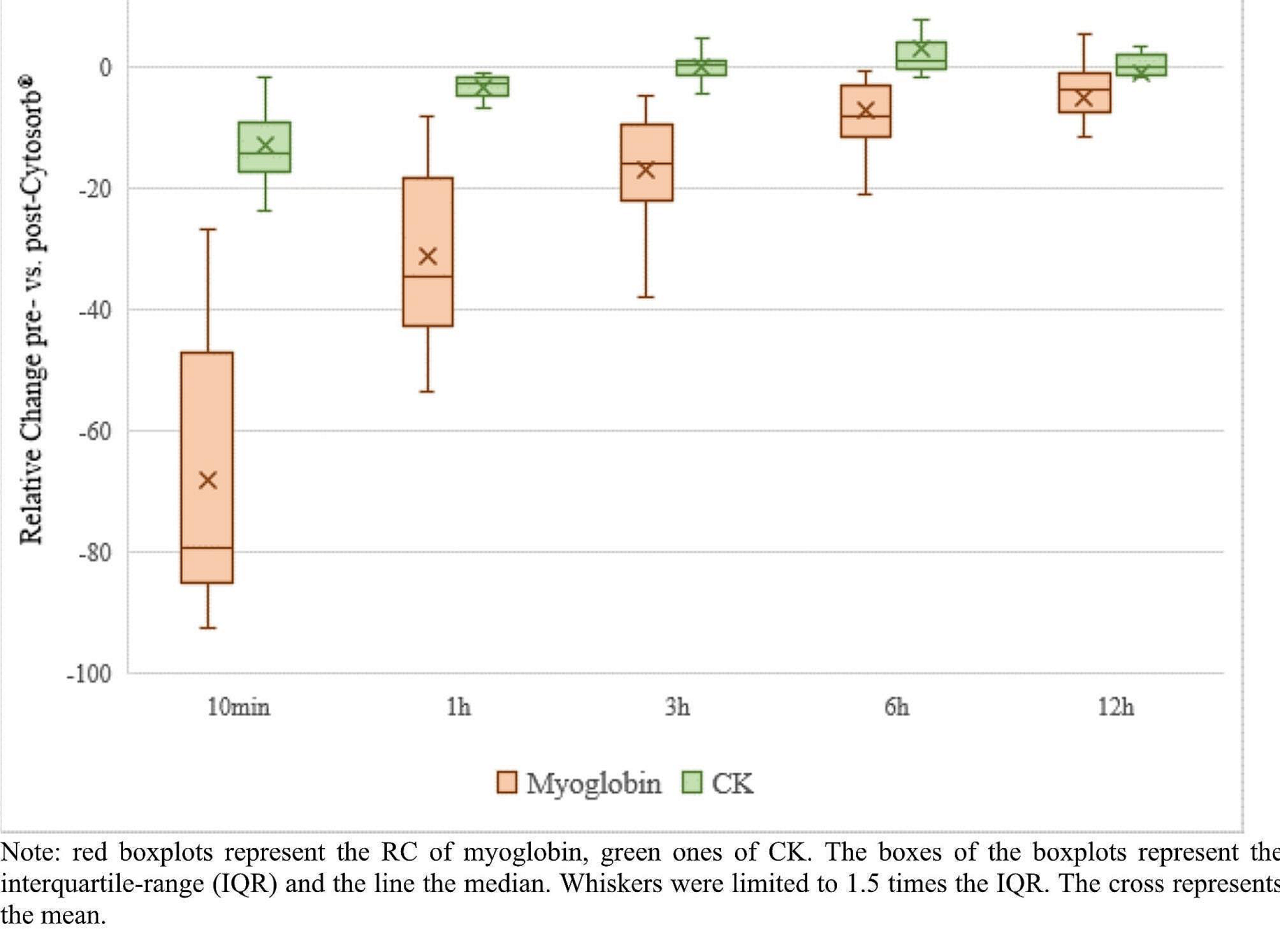
Relative Change (%) of myoglobin and CK due to Cytosorb®. *Note * red boxplots represent the RC of myoglobin, green ones of CK. The boxes of the boxplots represent the interquartile-range (IQR) and the line the median. Whiskers were limited to 1.5 times the IQR. The cross represents the mean

The median (IQR) RC of myoglobin at the above-mentioned time points was − 79.2% (-85.1; -47.1%), -34.7% (-42.7;-18.4%), -16.1% (-22.1; -9.4%), -8.3% (-7.5; -1.3%), and − 3.9% (-3.9; -1.3%), respectively. Further, the median RC of CK at the defined time points was − 14.3% (-14.3; -9.1%), -2.8% (-5.0; -1.7%), 0.1% (-1.6; +1.0%), 1.0% (-0.4; + 3.9%) and 0.0% (-1.5; +1.9%), respectively. Figure 3 shows the change of RC of myoglobin and CK during the application of CS.

### Plasma clearance of myoglobin during CS application

The median (IQR) plasma clearance of myoglobin ten minutes after the application of CS was 64.0 ml/min (58.6; 73.5 ml/min) and decreased rapidly to 29.1 ml/min (26.5; 36.1 ml/min), 16.1 ml/min (11.9; 22.5 ml/min), 7.9 ml/min (5.5; 12.5 ml/min), and 3.7 ml/min (2.4; 6.4 ml/min) after one, three, six, and twelve hours, respectively.

Since the median plasma myoglobin concentration at baseline was 56,984 ng/ml, we divided the cohort into patients with a baseline myoglobin concentration below (Group 1: *n* = 10; 7 CVVHD, 3 CVVHDF) and above the median value (Group 2: *n* = 10; 7 CVVHD, 3 CVVHDF). Groups 1 and 2 had an initial median (IQR) myoglobin concentration of 11,691 ng/ml (9664; 38,643 ng/ml) and 99,466 ng/ml (84,332; 142,420 ng/ml), respectively. Table 2 shows the median myoglobin plasma clearances for both groups at all measured time points.

**Table 2:** Myoglobin plasma clearance of the two groups

	10 min	1 h	3 h	6 h	12 h
Median (IQR) myoglobin plasma clearance (ml/min) Group 1	64.5 (59.8; 89.3)	33.5 (28.6; 44.2)	19.4 (15.4; 25.1)	10.1 (8.0; 14.9)	6.0 (2.6; 8.1)
Median (IQR) myoglobin plasma clearance (ml/min) Group 2	62.9 (57.0; 69.5)	26.8 (20.6; 29.8)	14.4 (10.3; 17.1)	5.9 (3.0; 7.4)	3.0 (0.6; 5.9)

**Fig. 4:**
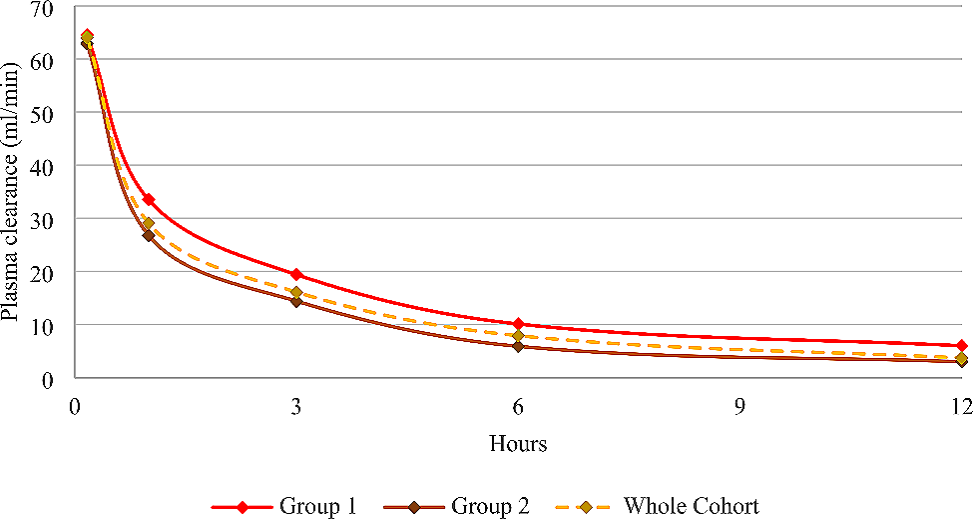
Myoglobin plasma clearance of group 1 (red), 2 (brown), and the whole cohort (yellow)

## Discussion

Severe rhabdomyolysis accompanied with high levels of myoglobin is a critical condition leading to acute kidney injury and in consequence to the potential need of renal replacement therapy [5, 7]. Since myoglobin in particular appears to be the main cause of kidney damage in rhabdomyolysis, one therapeutic approach is the extracorporeal removal of myoglobin [1]. In addition to causal and supportive therapy, various modalities of RRT as well as different dialyzers (high-/medium-cutoff) or the Cytosorb® adsorber cartridge for myoglobin elimination have been evaluated in the past [14–16, 22]. Considering there are few analyses elaborating the use of CS in rhabdomyolysis, especially in terms of adsorption capacity and saturation kinetics, this study was performed.

CS is approved for use in rhabdomyolysis and its ability to eliminate myoglobin in patients with rhabdomyolysis has been previously demonstrated [19, 23]. This is consistent with our results, showing a significant extracorporeal reduction of myoglobin at all time points. Although effective myoglobin clearance occurs in the first three hours after CS initiation, our analysis showed a rapid decline in myoglobin clearance after three to six hours. This reflects the patients’ plasma myoglobin concentration, which drops after six hours but rises to concentrations equal to or greater than baseline after twelve hours. These results implicate rapid saturation of CS, leading to inefficient adsorption for the remaining time of usage. Furthermore, brick saturation of CS is even more recognizable when considering patients with very high myoglobin concentrations. As we divided our cohort in two groups (initial myoglobin < and > 56,894 ng/ml (median baseline myoglobin concentration)), significantly lower clearance at one, three and six hours can be observed in the group with higher baseline myoglobin concentrations.

Recently, Albrecht et al. compared the myoglobin clearance of a high-cut-off dialyzer alone (*n* = 4) and in combination with CS (*n* = 4). They also describe early CS saturation with a median relative reduction of only 18% after two hours, which is quite similar to our results [23]. On the contrary, Albrecht et al. describe no difference in the velocity of saturation of CS in patients with a high baseline myoglobin concentration, indeed there was solely one patient with myoglobin levels > 30.000 ng/ml and only four patients in the entire cohort [23]. Nevertheless, there is a rapid saturation of CS not only for myoglobin, but also for other substances as bilirubin and bile acids [24]. Dilken et al. therefore changed the CS after twelve hours as they noticed a saturation with ongoing rhabdomyolysis in their patient, which lead to a further decrease in myoglobin [18].

High cut-off dialyzers such as EMiC®2 are also suitable and approved for myoglobin elimination. Weidhase et al. reported significantly higher myoglobin plasma clearance in high cut-off CVVHD (EMiC®2) compared to high-flux CVVHDF (Ultraflux® AV 1000 S). The advantage was a constant myoglobin plasma clearance of approximately 8 ml/min over 24 h [15]. In contrast, we observed a way higher myoglobin plasma clearance by CS in the first hour of application, which rapidly decreased to < 8 ml/min after only six hours. Comprising, a shorter change interval should be discussed, for example after three to six hours instead of after twelve to twenty-four hours, as the fabricator advises [25]. However, the side-effects of CS application, such as possible adsorption of anti-infective agents, a reduction in platelets count, and decrease in albumin concentration, as well as higher costs due to more frequent changes should also be taken into account [26–32].

Apart from the elimination properties of the different devices, the question of clinical benefit should be addressed. Gräfe et al. arises the question whether myoglobin elimination with CS integrated into RRT might lead to a faster kidney recovery compared to RRT alone in a propensity score matched cohort. They observed a significant higher probability of kidney recovery and significant lower myoglobin levels in patients receiving CS therapy [33]. Most recently, de Fallois et al. compared conservative management of rhabdomyolysis (without RRT) with extracorporeal therapies using different modalities, dialyzers, and an adsorber [34]. There were no significant differences in myoglobin reduction between the RRTs or between RRT and conservative treatment, but no information was given on the changing interval of CS [34]. In fact, patients without the need of RRT had the highest rate of myoglobin reduction, so preserving patients´ own renal function should be the primary goal in patients with rhabdomyolysis [34]. Therefore, CS therapy as a stand-alone device should be discussed in the future to perhaps prevent the kidney damaging effects of myoglobin. However, currently no data exist on the use of CS as a stand-alone device in the context of rhabdomyolysis and future studies would be desirable. Of course, the risks of extracorporeal procedures such as catheter infection, bleeding and thrombosis must be considered as well as device-associated side effects and complications [35, 36].

Notwithstanding that CK with a molecular weight of approximately 80 kDa should lie beyond the adsorption spectrum of CS (up to 60 kDa), extracorporeal elimination of CK was measured. There was a significant extracorporeal decrease of CK after ten minutes and one hour. However, the RC of CK was already considerably lower than the myoglobin clearance immediately after the installation of CS, and dropped to almost zero after three hours. Dilken et al. and Moresco et al. both describe a successful reduction of plasma concentration of myoglobin and CK in a case report [18, 37]. Also Albrecht et al., who as well analyzed extracorporeal samples, showed a short lasting but present relative reduction for CK [23]. Therefore, a higher adsorption spectrum for CS than previously assumed should be considered and verified, especially with regard to further side effects.

To the best of the authors´ knowledge, this is one of the first prospective studies to quantify extracorporeal myoglobin and CK adsorption of the CS cartridge itself. In summary, there is a significant extracorporeal elimination of myoglobin, but a rapid saturation of CS leads to an ineffective adsorption after three to six hours. An even faster decline of the myoglobin clearance was detected, especially in patients with very high myoglobin levels. These findings are important in order to improve the efficacy of the CS used in patients with rhabdomyolysis in clinical practice. Early change of adsorber seems to be crucial to avoid ineffective adsorption due to saturation, especially in patients with very high myoglobin levels. Therefore, serum myoglobin concentrations could be monitored at shorter intervals during CS therapy in order to respond to rising myoglobin levels. However, with more frequent adsorber changes, clinicians should be aware of an increased risk of side effects as adsorption of anti-infective agents, a reduction in platelets count, and decrease in albumin concentration [26–32]. Also, no significant reduction in CK can be expected from CS therapy.

This study has several limitations. First, the cohort of 20 patients appears to be small and inhomogeneous since reasons for rhabdomyolysis were quite diverse. However, despite the various causes, all patients have comparatively very high myoglobin levels and this is the largest prospective study in this field to date. In addition, the study objective was achieved with the patients included in the exploratory study. Second, both CVVHD and CVVHDF were used as dialysis modalities, yet this should have no impact on the myoglobin elimination of CS itself as the samples were collected extracorporeally right before and after CS and therefore unattached to possible myoglobin elimination by the dialyzer. Furthermore, some patients showed minor urine production during CS application, but the effect on the plasma myoglobin should be negligible for output of this magnitude. Since this study focused on elimination and saturation kinetics, the influence of CS on the outcome of the patients remains uncertain. Therefore, future randomized controlled clinical trials are needed to demonstrate the benefit of CS or other devices for myoglobin removal (e.g. EMiC®2) on the outcome of patients with severe rhabdomyolysis.

## Conclusion

The Cytosorb® adsorber effectively eliminates myoglobin. However, the adsorption capacity decreases rapidly after about three hours, resulting in a reduced elimination. Early change of the adsorber in patients with severe rhabdomyolysis, especially in patients with very high myoglobin levels, might increase the efficacy. Therefore, and in order to investigate a clinical benefit of the therapy, further randomized controlled studies are necessary.

### Electronic supplementary material

Below is the link to the electronic supplementary material.

Supplementary Material 1

## Data Availability

All data generated or analyzed during this study are included in this published article [and its supplementary information files].

## Abbreviations

AKI: Acute kidney injury
ARDS: Acute respiratory distress syndrome
BMI: Body mass index
CK: Creatine kinase
CRP: C-reavtive protein
CS: Cytosorb®
CVVHD(F): Continuous veno-venous hemodialysis/hemodiafiltration
ICU: Intensive care unit
LDH: Lactate dehydrogenase
RC: Relative Change
RRT: Renal replacement therapy
SAPS II: Simplified Acute Physiology Score
