# Supplementary material for: Myoglobin adsorption and saturation kinetics of the cytokine adsorber Cytosorb® in patients with severe rhabdomyolysis: a prospective trial
Source: Ann Intensive Care. 2024 Jun 22;14:96. doi: 10.1186/s13613-024-01334-x (PMC11192705; doi:10.1186/s13613-024-01334-x)
Supplement: Supplementary file 1 — Supplementary Material 1 [file 13613_2024_1334_MOESM1_ESM.docx]

**Supplemental Files**

Table S1: Plasma concentrations of myoglobin (ng/ml) and CK (U/l) before initiation, six and twelve hours after CS

| Patient | Myoglobin (ng/ml) 0h | Myoglobin (ng/ml) 6h | Myoglobin (ng/ml) 12h | CK (U/l)  0h | CK (U/l)  6h | CK (U/l)  12h |
| --- | --- | --- | --- | --- | --- | --- |
| 1 | 7157 | 5238 | 4794 | 857 | 993 | 831 |
| 2 | 183419 | 164995 | 145920 | 59375 | 58355 | 59525 |
| 3 | 11397 | 6369 | 7879 | 756 | 537 | 617 |
| 4 | 10499 | 7385 | 8553 | 14517 | 13504 | 11862 |
| 5 | 38258 | 38689 | 52189 | 9316 | 12136 | 17543 |
| 6 | 39796 | 23436 |  | 26682 | 23363 |  |
| 7 | 84300 | 74133 |  | 10500 | 10808 |  |
| 8 | 112738 | 137452 | 170130 | 12111 | 15293 | 18275 |
| 9 | 106009 | 100790 | 135158 | 19281 | 22771 | 33213 |
| 10 | 91541 | 54385 | 57178 | 39617 | 35942 | 32663 |
| 11 | 4195 | 4134 |  | 309,01 | 356,36 |  |
| 12 | 12072 | 9359 | 10848 | 644,6 | 533,52 | 555,77 |
| 13 | 64252 | 41560 | 38130 | 27226 | 21182 | 16858 |
| 14 | 84342 | 65871 | 64058 | 23227 | 20489 | 20134 |
| 15 | 129227 | 64824 | 126558 | 43282 | 35921 | 60901 |
| 16 | 92922 | 76139 |  | 15126 | 19373 |  |
| 17 | 11159 | 7129 |  | 6647,4 | 5662,8 |  |
| 18 | 182000 | 109000 | 126000 | 118000 | 87763 | 85099 |
| 19 | 11985 | 10151 | 11274 | 1204 | 864 | 705 |
| 20 | 49536 | 32286 | 33472 | 24984 | 29983 | 30308 |

Table S2: Myoglobin concentrations (ng/ml) pre- and post-CS at the defined time points

| Patient | 10min pre (ng/ml) | 10min post (ng/ml) | 1h pre (ng/ml) | 1h post (ng/ml) | 3h pre (ng/ml) | 3h post (ng/ml) | 6h pre (ng/ml) | 6h post (ng/ml) | 12h pre (ng/ml) | 12h post (ng/ml) |
| --- | --- | --- | --- | --- | --- | --- | --- | --- | --- | --- |
| 1 | 7011 | 1689 | 5512 | 2840 | 5513 | 4401 | 5209 | 4644 | 4971 | 4576 |
| 2 | 136430 | 55128 | 133867 | 105539 | 166224 | 139563 | 149493 | 142666 | 151634 | 144264 |
| 3 | 6220 | 578 | 5425 | 2464 | 5362 | 3266 | 5964 | 4649 | 7683 | 6752 |
| 4 | 9002 | 1342 | 6839 | 3785 | 6762 | 4607 | 7807 | 6384 | 9084 | 8552 |
| 5 | 25674 | 16991 | 23726 | 20157 | 28510 | 25589 | 48835 | 47401 | 79898 | 61217 |
| 6 | 23664 | 10311 | 19060 | 15229 | 21769 | 20369 | 22480 | 20025 |  |  |
| 7 | 75797 | 22550 | 59262 | 44761 | 61642 | 55414 | 75133 | 72184 |  |  |
| 8 | 123546 | 88988 | 116213 | 105650 | 149896 | 141111 | 135233 | 166349 | 222708 | 232588 |
| 9 | 90386 | 49268 | 79752 | 64465 | 83266 | 74181 | 108565 | 104058 | 142065 | 138859 |
| 10 | 65308 | 38444 | 54976 | 45755 | 56790 | 50268 | 58360 | 50952 | 60316 | 52661 |
| 11 | 4133 | 1823 | 3553 | 2382 | 3937 | 3224 | 4344 | 4096 |  |  |
| 12 | 10213 | 1412 | 8828 | 5213 | 8048 | 6230 | 9811 | 8646 | 11145 | 10194 |
| 13 | 57121 | 32644 | 49017 | 41585 | 39276 | 36896 | 40209 | 39526 | 44520 | 43942 |
| 14 | 70635 | 9797 | 69981 | 42435 | 59176 | 51221 | 64136 | 58500 | 63415 | 58222 |
| 15 | 101078 | 15676 | 46421 | 23803 | 35704 | 24209 | 64318 | 57971 | 130366 | 125705 |
| 16 | 88519 | 13109 | 67577 | 46671 | 62681 | 53864 | 77199 | 67859 |  |  |
| 17 | 10852 | 713 | 8776 | 4739 | 6819 | 5229 | 9070 | 7759 |  |  |
| 18 | 162000 | 22879 | 99943 | 61552 | 104000 | 82974 | 118000 | 109000 | 127000 | 127000 |
| 19 | 11152 | 1112 | 8876 | 5466 | 9376 | 7372 | 10823 | 8907 | 10990 | 10724 |
| 20 | 45256 | 6658 | 34470 | 20740 | 32920 | 24320 | 30284 | 27809 | 32767 | 31584 |

Table S3: CK concentrations (U/l) pre- and post-CS at the defined time points

| Patient | 10min pre (U/l) | 10min post (U/l)) | 1h pre (U/l) | 1h post (U/l) | 3h pre (U/l) | 3h post (U/l) | 6h pre (U/l) | 6h post (U/l) | 12h pre (U/l) | 12h post (U/l) |
| --- | --- | --- | --- | --- | --- | --- | --- | --- | --- | --- |
| 1 | 930 | 819 | 948 | 901 | 1032 | 1017 | 1011 | 1045 | 888 | 889 |
| 2 | 52319 | 45655 | 54775 | 52928 | 58578 | 55372 | 58302 | 59679 | 60984 | 61641 |
| 3 | 584 | 470 | 634 | 596 | 516 | 491 | 544 | 536 | 651 | 639 |
| 4 | 13811 | 11531 | 13388 | 13034 | 14365 | 14275 | 14235 | 14013 | 12001 | 11810 |
| 5 | 10339 | 9709 | 11525 | 11276 | 12559 | 12751 | 15378 | 15284 | 28513 | 23697 |
| 6 | 24136 | 21359 | 24361 | 23502 | 24400 | 24458 | 21134 | 23616 |  |  |
| 7 | 9777 | 8179 | 9376 | 9131 | 10010 | 9803 | 11305 | 11376 |  |  |
| 8 | 14845 | 14381 | 15633 | 15318 | 18021 | 18459 | 15541 | 19879 | 25605 | 26162 |
| 9 | 19667 | 17700 | 20514 | 19844 | 20692 | 20445 | 25077 | 24646 | 35577 | 34548 |
| 10 | 35726 | 34789 | 38610 | 36781 | 36648 | 36330 | 37753 | 34441 | 33450 | 30311 |
| 11 | 326,27 | 292,42 | 334,28 | 307,89 | 350,99 | 340,56 | 375,17 | 370,12 |  |  |
| 12 | 640,35 | 548,7 | 603,32 | 561,55 | 476,25 | 462,76 | 552,9 | 551,17 | 567,63 | 572,72 |
| 13 | 31510 | 29897 | 31279 | 30422 | 23763 | 23129 | 22190 | 23663 | 19498 | 19905 |
| 14 | 21696 | 17418 | 21772 | 20408 | 20138 | 20156 | 20703 | 20788 | 20530 | 20022 |
| 15 | 39251 | 31977 | 35745 | 33631 | 34423 | 34220 | 36302 | 35322 | 63750 | 63130 |
| 16 | 15355 | 12538 | 15700 | 14616 | 16950 | 17552 | 21067 | 20913 |  |  |
| 17 | 8691,4 | 6535 | 8496,9 | 8160 | 8515,3 | 8498,6 | 7586 | 7822,4 |  |  |
| 18 | 114000 | 91978 | 102000 | 99291 | 92158 | 89931 | 90151 | 91781 | 90388 | 90120 |
| 19 | 1183 | 973 | 1096 | 1057 | 1045 | 1035 | 852 | 845 | 683 | 689 |
| 20 | 24279 | 19901 | 25231 | 24189 | 28174 | 28074 | 29667 | 29822 | 31371 | 30953 |
